# Exploring the Effectiveness and Potential Pharmacological Mechanism of Minocycline for Spinal Cord Injury through Meta-Analysis and Network Pharmacology

**DOI:** 10.2174/1570159X23666250313104646

**Published:** 2025-03-14

**Authors:** Cai-wei Hu, Zhuo-yao Li, Ke Zhu, Yu-xiang Dai, Cheng Zhang, Yue-li Sun, Qi Shi, Xue-jun Cui, Min Yao

**Affiliations:** 1 Spine Disease Institute, Longhua Hospital, Shanghai University of Traditional Chinese Medicine, Shanghai, 200032, China;; 2 Key Laboratory of Theory and Therapy of Muscles and Bones, Ministry of Education, Shanghai University of Traditional Chinese Medicine, Shanghai, 200062, China

**Keywords:** Minocycline, spinal cord injury, systematic review, antioxidant, anti-inflammation, anti-apoptosis

## Abstract

Spinal cord injury (SCI) has a catastrophic impact and lifelong functional incapacity on patients. Recent research has demonstrated the anti-inflammation and neuroprotection of minocycline, which were advantageous for treating disorders having an inflammatory foundation, including SCI. This study summarized the antioxidant, anti-inflammation, and neuro-restoration of minocycline. PubMed, Web of Science, Embase, and Chinese database were explored from their origin date to July 2022. Data extraction, methodological quality assessment, and study selection were conducted by 2 reviewers. Twenty-four studies were ultimately included. Overall, minocycline improved motor recovery after SCI, with Basso Beattie Bresnahan (BBB) scores in the treated group from the first week (15 studies, *n* = 378; MD = 2.34; 95% Confidence interval (CI), 1.31-3.36; *p* < 0.00001) to the fourth week (14 studies, *n* = 346; MD = 3.15; 95% Confidence Interval (CI), 2.07-4.23; *p* < 0.00001). Subgroup analysis showed function recovery was related to the mode of drug dose, animal race, and article quality. Network pharmacology identified 100 minocycline-related targets and 6720 SCI-related targets. Heat Shock Protein 90 Alpha Family Class A Member 1(HSP90AA1), Serine/Threonine kinase 1(Akt1), Steroid Receptor Coactivator (SRC), Epidermal growth factor receptor (EGFR) and Catenin (Cadherin-Associated Protein)-Beta 1 (CTNNB1) were key targets. 20 pathways were identified, including PI3K/Akt, MAPK and chemokine signaling pathway. Finally, molecular docking results showed B-cell CLL/lymphoma 2 (BCL2-6), CTNNB1, HSP90AA1, plasminogen activator urokinase (PLAU), and α protein kinase C alpha (PRCAKA) bound to minocycline better. This article concluded that minocycline was effective in treating SCI by improving neurological recovery and inhibiting oxidative stress, apoptosis, and inflammation.

## 1 INTRODUCTION

Spinal cord injury (SCI) has an obliterating influence on the physical, financial, and psychosocial well-being of patients and their family members due to its long-term utilitarian incapacity [1]. According to previous research, it has an incidence of 10.4-83 cases per million people every year globally, with a high rate of incidence and a high societal expense [2]. The disease not only imposes a huge financial burden on patients but also puts a heavy burden on the whole society. On the basis of the severity of the injury, the whole life cost of curing an SCI person can range from 500,000 to 2 million dollars (serious injuries result in increased disability and costs). In the USA, the total annual direct expenditures of looking after SCI people surpass 7 billion dollars [3, 4]. Despite extensive research on the pathophysiology of SCI, neuroprotection, and anti-inflammation, non-surgical treatments have had limited clinical application by far. Current conservative treatment strategies for SCI mainly include drug administration [5].

Minocycline, or 7-dimethylamino-6-dimethyl-6-deoxyte-tracycline, has been used for over 30 years and is licensed for treating rheumatoid arthritis, several sexually transmitted infections, and acne vulgaris [6]. Minocycline's neuroprotective effects were initially demonstrated in the ischemia animal model by Yrjanheikki *et al*. in 1998 [7]. Minocycline improved CA1 pyramidal neuron survival and totally blocked ischemia-induced microglia activation and the development of NADPH-diaphorase reactive cells. Also this experiment also revealed a decrease in mRNA induction of interleukin-1β-converting enzyme. Moreover, minocycline inhibited oxidative stress and decreased ischemic neuron damage both *in vitro* and *in vivo* [8]. It was discovered that minocycline may have protective benefits against glutamate-induced neuronal injury because it inhibited the growth and activation of microglia [9]. In short, these studies illustrated minocycline's anti-inflammation, neuroprotection, and anti-oxidative stress, which were thought to be advantageous for treating disorders having an inflammatory base, such as SCI. However, several studies have shown that minocycline lacked neuroprotection in a cervical SCI model and showed little improvement in functional recovery. Both minocycline and simvastatin administration showed limited improvement in behavioral tests compared to placebo controls [10]. Additionally, all groups performed similarly on the von Frey sensory test, cylinder rearing test, and horizontal ladder test. There were no remarkable differences in corticospinal and rubrospinal sprouting, white and gray matter sparing, or functional recovery between the administration groups in the contusive model. Furthermore, pre-clinical research on neuroprotective therapies has included minocycline so far, which was administered non-invasively for acute SCI [11]. To sum up, the treatment effect of minocycline for SCI remained questioned.

In this research, a meta-analysis was conducted to study the effectiveness of minocycline against SCI. A network pharmacology approach was conducted to investigate minocycline's possible pharmacological mechanisms against SCI. The binding mode of minocycline and its protein targets was then determined through a molecular docking approach.

## 2 METHODS

### 2.1 Meta-analysis to Assess the Effectiveness of Minocycline Against SCI

#### 2.1.1 Searching Strategy

Literature was retrieved from 5 databases, including PubMed, Web of Science, Embase, China National Knowledge Infrastructure (CNKI), the VIP information resource integration service platform (VIP), Chinese Biomedical Literature Database (SinoMed), and Wanfang Data knowledge service platform (Wanfang Data) with the searching keywords “minocycline”, “7-dimethylamino-6-dimethyl-6-deoxytetracycline”, “spinal cord injury”, “spinal cord injuries”, “rat” and “rats”. Moreover, the references of included studies and considerable conference papers were examined for supplementary relevant information. The searching date ranged from the origin to June 2022. The literature search had no language restrictions and was limited to rat experiments.

#### 2.1.2 Study Selection

Two reviewers (CWH and MY) independently read all articles during the primary screening, focusing on the title and abstract. After that, the full text was scrutinized for a second screening. If disagreements appear, they will be resolved through discussion with a third party (XJC).

The criteria for participants, interventions, comparisons, outcomes, and study design (PICOS) were used as inclusion criteria: (1) Rats of any age or gender were used in experiments involving traumatic SCI, such as bruising, impactor injury, crush, and compression trauma; (2) Intervention: There were no restrictions on the minocycline dosage, treatment method, duration of therapy, or follow-up time (3) Comparison: The control groups included filtered saline, vehicle, or no treatment; (4) Outcomes: The primary outcome was chosen to be the Basso, Beattie, and Bresnahan (BBB) score [12, 13]. Malondialdehyde (MDA) and superoxide dismutase (SOD) were selected as oxidative stress injury indicators. Glial fibrillary acidic protein (GFAP) and interleukin-6 (IL-6) levels were regarded as inflammation markers. In the final mechanism analysis, other outcome measurements were also taken into account: (5) Study design: A comparison between minocycline and control group in SCI rats.

Studies that were disqualified had one or more of the following aspects: (1) SCI rat models with nontraumatic injury or peripheral nerve injury were excluded. (2) Animal experiments conducted with genetically modified rats were excluded. (3) Minocycline in combination with other intervention treatments was not considered. (4) Clinical case reports, duplicate articles, reviews, articles without animal experiments, and irrelevant studies, including minocycline treating neuropathic pain, were excluded.

#### 2.1.3 Data Extraction

Two reviewers separately extracted the information from the papers. Data were gathered from each paper, including information about the author and the year of articles published, the sort of models and group compared, the therapeutic dose and injection approach of minocycline, and the outcome evaluation. The mean ± standard deviation (SD) of each result was gathered for the pooled analysis. The discussion was raised when disagreements appeared. Whenever necessary, the opinion of a third reviewer was requested. In situations where there was a lack of data or insufficient data, the authors were contacted *via* email and sought for numerical data.

#### 2.1.4 Bias Risk Assessment for Included Articles

The Collaborative Approach to Meta-Analysis and Review of Animal Data from Experimental Stroke (CAMARADES) 10-item checklist was utilized for evaluating the quality of articles by 2 separate reviewers [14]. The assessment included the following parts: (1) The journal was peer-reviewed or not; (2) whether temperature control was mentioned in the article or not; (3) animals were randomly allocated or not; (4) blind established model was conducted or not; (5) outcome assessment was blinded or not; (6) anesthetics used was without intrinsic neuroprotection or not; (7) animal model (with diabetes, elderly or hypertensive) was healthy or not; (8) calculation of sample size was demonstrated in the article or not; (9) compliance of animal welfare regulations was declared in the article or not; (10) there is possible conflicts of interest in the article or not.

Bias assessment results were classified as high, low, or unclear. The symbol “+” represented a low risk, also recorded as a score point. The symbol “-” meant high risk, while the “?” was considered “unclear,” which meant that the article did not mention this item.

#### 2.1.5 Statistical Analysis

Statistical analysis was implemented through Review Manager 5.4 (RevMan 5.4). Compared with SCI groups, data from all minocycline groups were pooled. The weighted mean difference (WMD) or standardized mean difference (SMD) and 95% confidence interval (CI) were determined using RevMan 5.4. Using a random effect model, the cumulative effect sizes were estimated. Q statistics and I2 statistics were used to quantify heterogeneity. Statistical significance was defined at *p* < 0.05. The fixed effect model was utilized, when heterogeneity was not remarkable, namely *p* > 0.1; I^2^ ≤ 50%; when *p* ≤ 0.1; I^2^ > 50%, a random effect model was taken into consideration.

### 2.2 Network Pharmacology and Molecular Docking Approach

#### 2.2.1 Screening of Target Proteins and the Conduction of Protein-protein Interaction (PPI) Network

Through the PubChem database (https://pubchem.ncbi.nlm.nih.gov/), the spatial data file format (SDF) of minocycline could be collected. Swiss Target Prediction (http://www.swisstargetprediction.ch/) was used for the prediction of each target while the species was chosen as *Homo sapiens*. After obtaining the target proteins, the UniProt database (https://www.uniprot.org/) was utilized to transfer targets to standard target symbols.

SCI-related target genes were screened from the Online Mendelian Inheritance in Man (OMIM) (https://omim.org/) and GeneCards (https://www.genecards.org/) databases. Searching expressions were “spinal cord injury” and “spinal cord injuries”. So far, all target genes associated with minocycline and SCI were collected while the duplicated genes were removed. Subsequently, through online Venn mapping software (https://bioinfogp.cnb.csic.es/tools/venny/), the overlapping target genes between minocycline-related targets and SCI-related genes were identified and visualized.

In order to explore the further interactions among overlapping genes, the STRING 11.0 database (https://string-db.org/) was used. The species was set as “*Homo sapiens*” after selecting “Multiple proteins” and kept the default values for other parameters. The obtained protein interaction results were stored in TSV format. Then, the file was imported into Cytoscape software to visualize the interactive network. The size and color shade reflected the importance of nodes, while the thickness of the edge represented the combination score.

#### 2.2.2 GO and KEGG Pathway Enrichment Analysis and Target-pathway (T-P) Network Building

To clarify the role of SCI targets resistance in gene function and understand the potential mechanisms of minocycline against SCI, Metascape (https://metascape.org/) was used for biological process (BP), molecular function (MF), cellular component (CC) enrichment and KEGG pathway analysis. Through the ImageGP website (http://www.ehbio.com/ImageGP/), KEGG pathways were visualized. Rich Factor refers to the number of differentially regulated genes within these metabolic pathways to the number of annotated genes within those pathways. A higher Rich Factor indicated a greater gene enrichment. The number of genes is reflected in the bubble size. A bigger bubble indicated a higher concentration of genes enriched in a particular pathway. Generally, the shade of the bubble is a measure of significance. In brief, a bigger and redder bubble represented a higher level of enrichment.

The top 20 KEGG pathways and related genes were screened out to build the target-pathway (T-P) network for minocycline against SCI in Cytoscape. Topological analysis was conducted on the constructed T-P network, and the size of nodes and shade of edges in the network were determined by ranking degree value.

#### 2.2.3 Molecular Docking

In AutoDock Vina, minocycline was added, along with the protein crystal structures from the Protein Data Bank (PDB) database (http://www.rcsb.org/pdb). After the water was removed, hydrogen addition and ligand root detection were conducted. Molecular docking of receptor proteins and minocycline was performed based on a genetic algorithm based on AutoDock Vina 1.1.2 software. Ultimately, the lowest docking score, namely the highest binding energy, was visualized using PyMOL (ver. 2.5.2) software based on the docking results.

## 3 RESULTS

### 3.1 Studies Selection

**Fig. (1):**
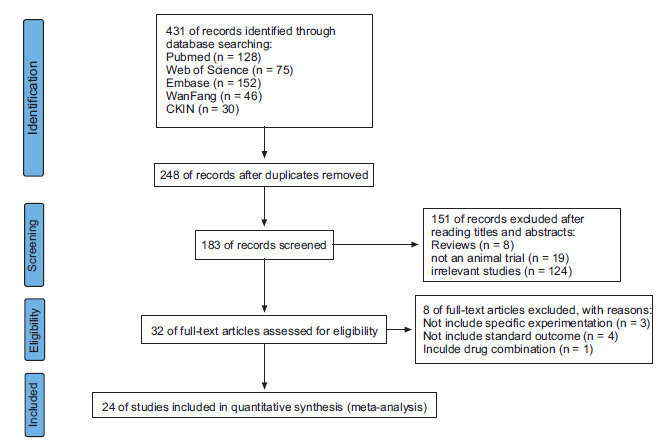
Summary of the literature search process.

A total of 431 relevant research literature were searched through a systematic search strategy. After eliminating duplicate articles and screening titles and abstracts, 32 studies were selected for full-text screening. Among the studies screened out in the full text, 3 studies did not include specific experiments, 4 studies did not include standard outcomes, and 1 study had a drug combination. Therefore, there were 24 studies in this article, and the detailed information mentioned above is shown in Table **1** (Fig. **1**) [15-38].

Among these 24 studies, the sample size of experimental animals ranged from 8-113. A model of spinal cord compression was used in 6 studies. The contusion injury model was used in 16 articles. One study conducted the descending aorta clipping and compression model at the same time. The transaction model was used once in all studies. The T9-T10 section was selected most frequently for modeling and included 14 articles [18, 21-24, 27, 29-31, 34-38]. One article used descending aorta injury, while one article did not mention the specific injured segment [17]. The positive controls include saline, tetracycline, and methylprednisolone. Doses of minocycline ranged from 6.4-93.75 mg/kg. Most studies opted for intraperitoneal administration of the minocycline. 2 articles chose oral administration, while one experiment was administrated through a vein. Also, one study did not mention its administration clearly.

### 3.2 Risk of Bias of Included Studies

The quality assessment of studies is shown in Table **2**. Peer-reviewed publications, random allocation, proper anesthetics, animal models, and animal welfare regulations declarations were emphasized in all articles. 17 of 24 studies’ scores exceeded 6 points after assessment, which implied that over 70% of researchers were good at methodology. Thirteen studies did not report temperature control. One study with descending aorta clipping and a compression model did not mention the sample size calculation in the article. Declaration of potential conflict of interests was mentioned in 8 studies. Fifteen studies did not contain a blinded assessment of the outcome.

### 3.3 The effect of Minocycline Intervention on BBB Scores in Rats with SCI

**Fig. (2):**
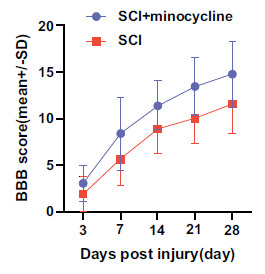
BBB score meta-analysis after SCI and the effect size line chart. **Abbreviations**: BBB, Basso, Beattie, Bresnahan; SCI, spinal cord injury.

Nineteen studies included behavioral data. The BBB score of rats treated with minocycline were significantly improved from day 7 (15 studies, *n* = 192, WMD = 2.34, 95% CI (1.31 to 2.26), *p* < 0.00001) and maintained an advantage since day 28 in comparison to the control group (14 studies, *n* = 176, WMD = 3.15, 95% CI (2.07 to 4.23), *p* < 0.00001) (Fig. **2**).

Subgroup analysis was performed on animal modeling, initial dosage, the choice of modeling segment, the mode of drug dose, placebo, race of modeling animals, and quality of the included articles. All groups showed no improvement in BBB scores except for the change in drug dose, the race of model animals, and the quality of the articles included. It was found that minocycline group with once-mode drug dose improved BBB scores better (14 d, 4 studies) [18, 19, 21, 24], MD = 4.07, 95% CI (2.76, 5.37), *p* = 0.0004; (Table **3**) than multi-mode drug dose statistically (14 d, 9 studies) [20, 22, 27, 29, 30, 34-37], MD = 1.74, 95% CI (1.27, 3.54), *p*< 0.00001; (Table **3**). Also, according to Table **3**, it could be concluded that a constant high dose of minocycline had a higher BBB score compared to the multi-mode administration group without a constant high administration dose, which may have a better therapeutic effect on SCI. The reason for this may be that a high concentration of minocycline can quickly reach the therapeutic serum concentration range and maintain the treatment effect for a longer time, benefiting the neurons better. Also, due to the changed dose of most experiments, the initial dosage of 45 mg/kg showed that it could improve the BBB score better. However, it only included 2 studies and one of the studies administered before modeling, which may affect the experiment result.

### 3.4 Biochemical Outcome

Ten studies (n = 554) had reported biochemical markers, including MDA, SOD, tumor necrosis factor-α (TNF-α) and IL-6. Malondialdehyde, a cytotoxic compound that causes the cross-linking of proteins, nucleic acids, and other living macromolecules, is the result of the oxidation of lipids induced by free radicals in living organisms. MDA is the byproduct of lipid oxidation, and its synthesis can exacerbate membrane damage by interfering with essential enzyme functions in mitochondria and the mitochondrial respiratory chain complex in vitro. Therefore, the amount of malondialdehyde represents the lipid peroxidation and cell damage in the body [39]. SOD has been linked to various disease states, including inflammatory illnesses, ischemia and reperfusion diseases, neurodegenerative illnesses, and more. It was a crucial signaling molecule in procedures including cell division and even served as a lipid peroxidation terminator [40]. MDA and SOD were used as indicators of oxidative stress in this research. One of the most significant cytokines-induced inflammation, TNF-α, contributed to the development of edema and vasodilation as well as leukocyte adherence to the epithelium by expressing adhesion molecules. It controlled blood coagulation, causing inflammation and oxidative damage [41]. IL-6 promoted acute phase reactions, hematopoiesis, and immune responses in reaction to infections and tissue injury, aiding in host defense [42].

MDA level decreased significantly after minocycline administration (3 studies, *n*=33; SMD=-4.78; 95% (CI), -7.55 to -2.01; *p*=0.002; Table **4**).

SOD level was reported in 2 studies which only contained the data of day 28 (2 studies, *n* =13; SMD=6.3; 95% (CI), 4.11 to 8.49; *p* =0.72; Table **4**). Notwithstanding the data's limitations, both investigations demonstrated that the treatment group and the control group differed from one another.

TNF-α expression was reported in 4 studies (4 studies, *n* = 43; SMD= -4.99; 95% (CI), -8.61 to -1.38; *p<*0.0001; Table **4**). The meta-analysis illustrated that there were variations between the minocycline and placebo groups in both studies.

IL-6 expression was also reported in 2 studies (2 studies, *n* = 25; SMD=-8.66; 95% (CI), -16.53 to -0.80; *p<*0.0001; Table **4**). The outcome revealed that there were variances between the drug group and the placebo group, as demonstrated by two articles.

### 3.5 Identification of Minocycline-related and SCI-related Genes and Analysis of the PPI Network of these Genes

**Fig. (3):**
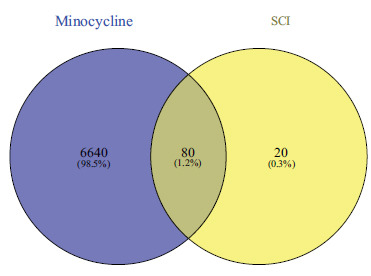
6720 SCI-related genes and 100 minocycline-related overlapped genes. SCI, spinal cord injury.

**Fig. (4):**
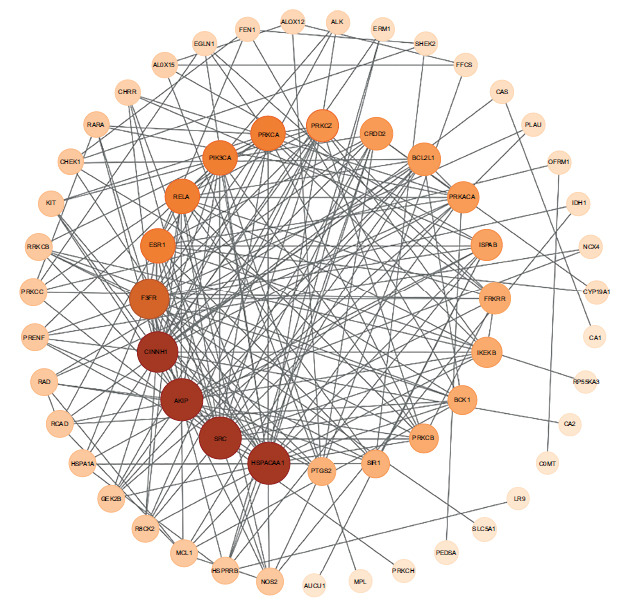
Protein-protein interaction network of overlapping target genes (target genes from minocycline treating spinal cord injury).

Six-hundred and seventy-two SCI-related target genes were found in the OMIM and GeneCards databases, while a total of 100 minocycline target genes were gathered from the PubChem database. Eighty overlapping genes between minocycline and SCI were shown by Venn diagrams (Fig. **3**). The top targets included Heat Shock Protein 90 Alpha Family Class A Member 1(HSP90AA1), Serine/Threonine kinase 1(Akt1), Steroid Receptor Coactivator (SRC), Epidermal growth factor receptor (EGFR) and Catenin (Cadherin-Associated Protein)-Beta 1 (CTNNB1) (Fig. **4**).

### 3.6 GO and KEGG Pathway Analysis of Overlapping Target Genes Treating SCI and Analysis of T-P Network against SCI

**Fig. (5):**
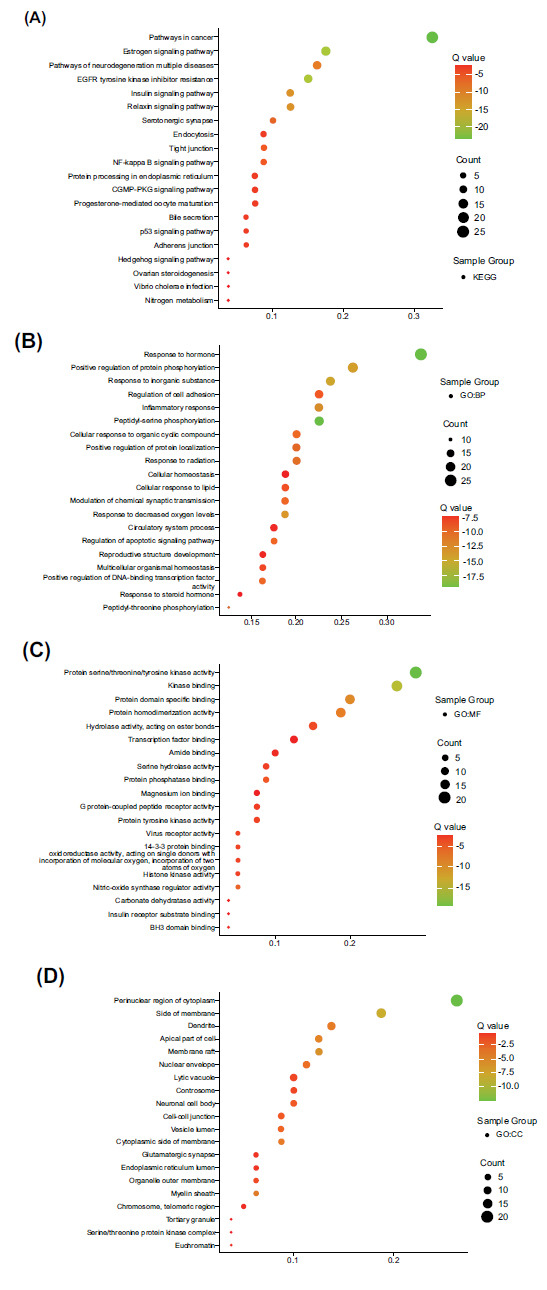
GO and KEGG pathway enrichment analysis of overlapping genes of minocycline and spinal cord injury (*p* < 0.05). (**A**) KEGG (**B**) Biological process. (**C**) Molecular function. (**D**) Cellular component. **Abbreviations**: GO, Gene ontology; KEGG, Kyoto encyclopedia of genes and genomes.

KEGG pathway enrichment demonstrated the top 20 signaling pathways. It can be concluded that neurodegeneration pathways, serotonergic synapse, NF-kappa B, p53, and hedgehog signaling pathways play a crucial role in treating SCI (Fig. **5A**).

The GO enrichment results indicated that minocycline might be responsive to hormones, positive regulation of protein phosphorylation, and inflammation response *via* protein serine/tyrosine kinase activity, kinase binding, protein domain specific binding in the perinuclear area of cytoplasm, side of the membrane, dendrite, apical part of cell and membrane draft exert the anti-SCI function (Figs. **5B**-**D**).

**Fig. (6):**
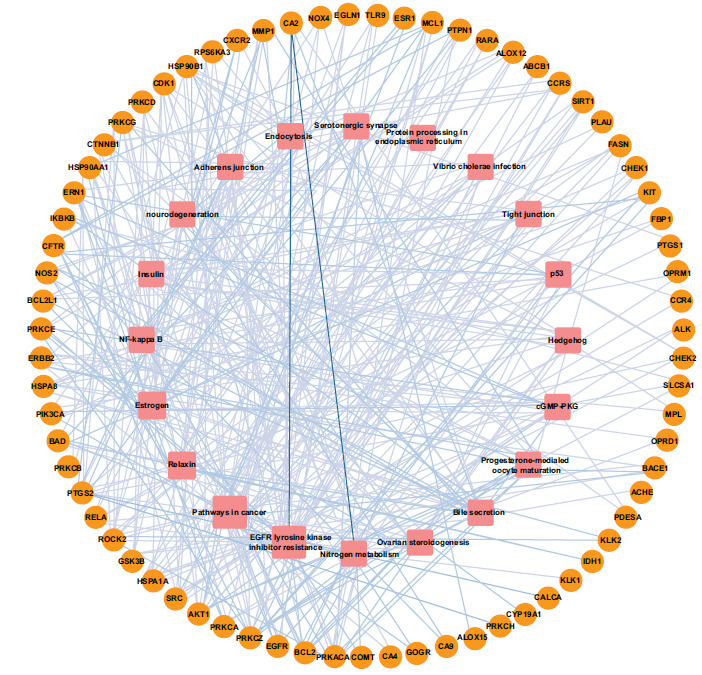
Target-pathway network of minocycline against spinal cord injury. The orange circles represented the overlapping genes from minocycline and spinal cord injury. Pink squares represented signaling pathways.

The T-P network was conducted to explore further relations between 20 KEGG pathways and its responding genes, which included 91 nodes and 283 edges (Fig. **6**). At the same time, the specific information of 20 KEGG pathways and related genes were shown in Table **5**. It can be concluded that phosphatidylinositol 3'-kinase-serine/threonine kinase (PI3K/Akt), Chemokine signaling pathway, and mitogen-activated protein kinase (MAPK) signaling pathway played an important role in treating SCI.

### 3.7 Molecular Docking

**Fig. (7):**
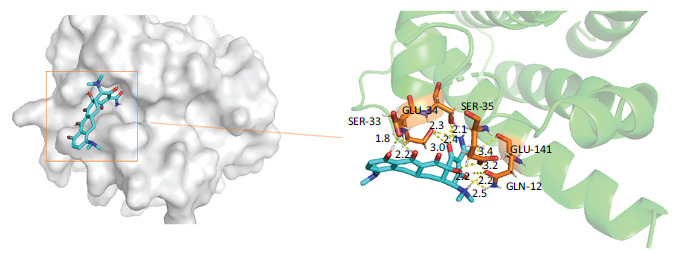
Interaction of minocycline with BCL2-6. BCL2-6, B-cell CLL/lymphoma 2.

**Fig. (8):**
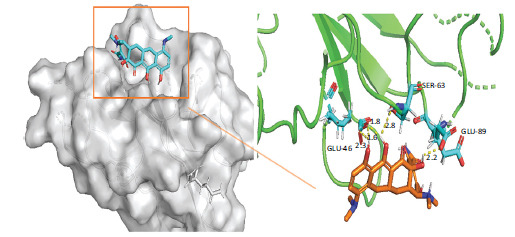
Interaction of minocycline with CTNNB1. CTNNB1, Catenin (Cadherin-Associated Protein)-Beta 1.

**Fig. (9):**
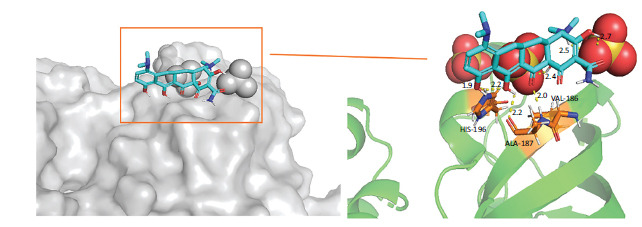
Interaction of minocycline with HSP90AA1. HSP90AA1, Heat Shock Protein 90 Alpha Family Class A Member 1.

**Fig. (10):**
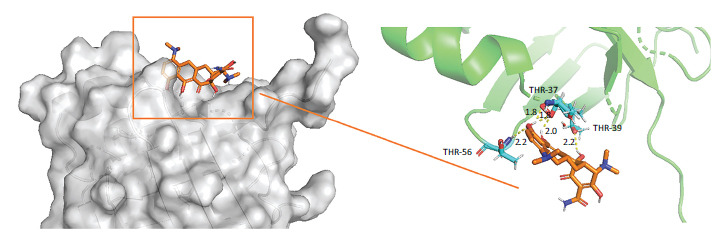
Interaction of minocycline with PLAU. PLAU, plasminogen activator urokinase.

**Fig. (11):**
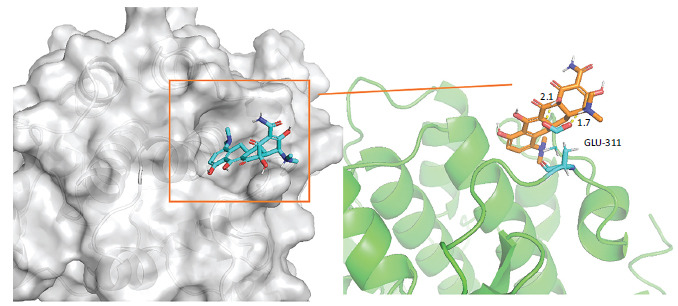
Interaction of minocycline with PRCAKA. PRCAKA, α protein kinase C alpha (PRCAKA).

A detailed explanation of the binding energy, as well as docking parameters, can be found in Table **6**. The binding modes between BCL2-6, CTNNB1, HSP90AA1, PLAU, and PRCAKA, as well as minocycline, were clearly exhibited (Figs. **7**-**11**). BCL2-6 and minocycline formed hydrogen bonds at SER-33, SER-35, GLU-34, GLU-141 and GLN-12. CTNNB1 and minocycline formed hydrogen bonds at GLU-47, GLU-82, and SER-63. HSP90AA1 and minocycline formed hydrogen bonds at VAL-186, ALA-187, and HIS-196. PLAU and minocycline formed hydrogen bonds at THR-57, THR-37 and THR-39. PRCAKA and minocycline formed hydrogen bonds at GLU-311.

It was discovered that molecular docking can replicate the binding properties of various molecules and proteins. Table **6** demonstrates the docking energy. These findings suggested that target genes can be successfully bound by minocycline.

## 4 DISCUSSION

### 4.1 The Summary of the Evidence

It is the initial meta-analysis of animal studies that shows that minocycline has been used to treat SCI and has been merged with a network pharmacology approach. BBB scores gradually increased over time and showed the most significant improvement at 7 d after minocycline treatment compared with the control group. Besides, minocycline considerably decreased the MDA level in the injured spinal cord and increased the SOD level in the minocycline group after SCI compared with the control group. TNF-α and IL-6 levels were also expressively decreased after minocycline administration. Hence, one may draw the conclusion that minocycline was crucial to the restoration of neurological function and the increase of antioxidant levels following SCI.

Also, in order to explore its efficacy and effective targets for action, the approach of network pharmacology and molecular docking was conducted. The top targets included HSP90AA1, Akt1, SRC, EGFR, and CTNNB1 through the PPI network. Based on T-P pathway analysis, PI3K/Akt, chemokine, and MAPK signaling pathways play an important role in treating SCI. Moreover, molecular docking results indicated that BCL2-6, CTNNB1, HSP90AA1, PLAU, and PRCAKA bind with minocycline tightly.

### 4.2 Molecular Mechanisms of Minocycline

SCI is caused by a primary trauma/ischemia-related lesion, which leads to secondary events [1]. The disabilities associated with SCI are caused by neuronal dysfunction. Vascular, inflammatory, and metabolic processes brought on by the original injury's secondary cascade SCI led to further disruptions in neuronal function [43]. Thus, minocycline could treat SCI mainly through anti-oxidative stress and anti-inflammation.

### 4.3 Protective effect of Minocycline Related to Anti-inflammation

A significant modulator of the progress of secondary injuries in SCI was inflammation. A few hours after SCI, the inflammatory response can quickly increase the number of mediators-induced inflammation (TNF-α, interleukin-1β (IL-1β), IL-6, and others), activating toll-like receptors (TLRs) in inflammatory cells, causing neuroinflammation and neurotoxicity [44]. NF-κB, lipopolysaccharide-induced tumor necrosis factor-alpha factor (LITAF), and Nur77 were instances of inflammation-associated transcription factors that have been demonstrated to be inhibited by minocycline through inhibiting the phosphorylation of p38 MAPK [45]. These transcription factors governed the production of leukocyte-recruiting chemokines and cytokines-induced inflammation, including monocyte-chemoattractant protein-1 (MCP-1), TNF-α, IL-1β and IL-6 [46]. This study demonstrated that the main mechanism of minocycline against SCI was the reduction of TNF-α and IL-6 levels, which were a key mediator of inflammation through the downregulation of p38 MAPK activation. Through the network pharmacology approach, it was shown that the PI3K/Akt signaling pathway was crucial in controlling the inflammatory response following spinal cord damage. Toll-like receptor 4 (TLR4) expression and the delivery of inflammatory substances like TNF-α and IL-6 in microglia were both attenuated by activated PI3K/Akt signaling [47]. Astrocytes had an intimate relationship with neurons in the central nervous system (CNS). It provided metabolic substrates for neurons, stabilized the extracellular ionic environment and pH, participated in the uptake of neurotransmitters, and helped maintain the integrity of the blood-brain barrier [48]. Therefore, it could be inferred that minocycline inhibited TLR4 expression through the activation of the PI3K/Akt signaling pathway, thereby reducing the level of inflammatory factors and ultimately achieving anti-inflammation after spinal cord damage.

Activated astrocytes released a range of pro-inflammatory cytokines (IL-1β) and chemokines, which induced neuronal sensitization. Therefore, reactive astrocytes play an important role in inflammation. It has also been identified that NF-κB mediated inflammatory pain in mice *via* chemokine production in spinal astrocytes [49]. The study found that minocycline inhibited NF-κB activation potentially through a reduction in IL-1β signaling [50]. Also, it was found that minocycline down-regulated NF-κB activity, in part, *via* the inhibition of transforming growth factor beta 1 [51]. Collectively, the current and previous results suggested that minocycline inhibited NF-κB activation to reduce inflammation.

### 4.4 Protective effect of Minocycline Related to Anti-oxidative Stress

Excessive synthesis of reactive species, including reactive oxygen (ROS) and nitrogen (RNS) species, as well as an unbalanced failure by the body's antioxidant enzyme systems, leads to the ruin of cellular molecules, lipids, protein, and gene products [39]. This study demonstrated that the primary mechanism by which minocycline prevented SCI was by limiting MDA levels while promoting SOD levels. SOD neutralized O_2_·^−^ to produce the neutral products H_2_O_2_ and H_2_O.

Lipids may become oxidized, and mitochondrial DNA (mtDNA) may become damaged as a result of Hydroxyl (·OH) and nitric oxide (NO·) radicals. Instead, protein oxidation and desulfurization were caused by peroxynitrile (ONOO·) [39]. Thus, the reduction of SOD leads to elevated oxidative stress, resulting in an imbalance in ROS production and antioxidant capacity in mitochondria, ultimately leading to oxidative mitochondrial damage, reduced mitochondrial function, and increased susceptibility to apoptosis. Additionally, following SCI, minocycline therapy boosted the activity of the free radical-scavenging enzymes glutathione peroxidase (GSH-Px) and SOD [50]. *Via* enzymatic and non-enzymatic processes, the body produced oxygen radicals that attacked polyunsaturated fatty acids in biological membranes, triggering lipid peroxidation and consequent formation of lipid peroxides, including MDA [39]. In this study, the result showed that minocycline treatment could decrease the level of MDA. An essential mechanism through which minocycline can slow the progression of secondary injuries is its anti-oxidative action. In brief, ROS/RNS production increased significantly after spinal cord damage, contributing to oxidative stress and causing extensive harm to nearby tissue as well as the cells that generate free radicals [33-52]. Minocycline can reduce reactive species production *via* previously discussed anti-oxidative stress mechanisms.

### 4.5 Protection of Minocycline against Apoptosis

By activating the TNF receptor 1 (TNFR1) and CD95 ligand (CD95L), respectively, CD95 and TNF-α may cause cell death in various cell types, including oligodendrocytes [53]. Moreover, microglia expressed CD95 and TNFR1, and when activated, these cells may produce CD95L and TNF-α [54, 55]. As a result, it was possible that activated microglia were the core of the damage cascade and were targets for treatment. When neurons were damaged, glutamate (Glu) was released into the extracellular space. High concentrations of glutamate triggered an excitotoxic cascade that could result in necrotic cell death. In turn, Glu could stimulate microglia, releasing inflammatory molecules, including TNF-α and CD95L, which activated apoptosis for a protracted length of time after injury. A previous study showed that in various types of CNS damage, including SCI, minocycline decreased microglial activation and apoptosis [34]. Also, more and more studies confirmed that treatment with minocycline can decrease apoptosis in oligodendroglia and neurons and enhance function [55].

The follicular lymphoma-related protein BCL-2 was critical in controlling apoptosis and promoting cell survival in response to various apoptotic triggers [57]. It has been identified that B-cell lymphoma/leukemia-2 (Bcl-2) may prevent the release of cytochrome c from mitochondria or bind to the apoptosis-activating factor (APAF-1) to inhibit apoptosis in several cell systems, including neurons [58]. Bcl-2 overexpression was proven to protect neurons from cell death caused by free radicals, hypoxia, or growth factor deficiency in numerous investigations [59-61]. In this study, molecular docking results indicated that BCL2-6 binds with minocycline tightly, which meant that minocycline could regulate the Bcl-2 family to achieve anti-apoptosis. Interleukin-10 (IL-10) was discovered to upregulate the anti-apoptotic proteins B-cell lymphoma 2 (Bcl-2), Bcl-2-associated X and B-cell lymphoma-extra-large (Bcl-xl), while downregulating the proteins induced apoptosis such as cytochrome c, caspase 3, and Bax [62]. The Binding of IL-10 and its receptor was activated by Jak1 and Tyk2 [63]. This would trigger the STAT1, STAT5, and STAT3 pathways after SCI. The pro-inflammatory cytokine generated by microglia was reduced through the indirect STAT3 pathway. The direct STAT3 route induced an anti-apoptotic cascade and was neuroprotective. The PI3K/Akt signaling pathway enabled IL-10 to protect neurons from excitotoxicity. In order to increase the transcription of the antiapoptotic proteins Bcl-2 and Bcl-xl, giving neurons the capacity to tolerate glutamate toxicity and traumatic injury, Akt restricted the release of cytochrome c and inhibited caspase 3 activation [64]. At the same time, IL-10 levels had been reported to rise in response to a number of exogenous therapies, including minocycline [65]. In short, it could be inferred that minocycline could increase the level of IL-10 to achieve anti-apoptosis.

**Fig. (12):**
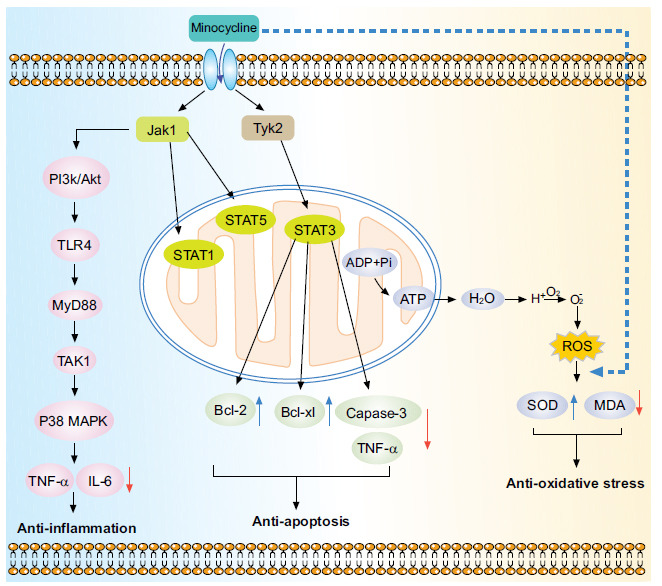
The mechanism for minocycline against SCI. **Abbreviations**: SCI, spinal cord injury; MINOR, minocycline receptor; TLR4, Toll-Like Receptor 4; MyD88, myeloid differentiation factor 88; Bcl-xl, B-cell lymphoma-xl; Bcl-2, B-cell lymphoma-2; Jak1, Janus activated kinase 1; Tyk2, tyrosine kinase 2; TAK1, transforming growth factor kinase 1; p38 MAPK, p38 mitogen-activated protein kinase; STAT1, signal transducer and activator of transcription 1; STAT3, signal transducer and activator of transcription 3; STAT5, signal transducer and activator of transcription 5; IL-6, interleukin-6; TNF-α, tumor necrosis factor alpha; ADP, adenosine diphosphate; ATP, adenosine-triphosphate; MDA, malondialdehyde; SOD, superoxide dismutase.

According to the pathology of SCI, network pharmacology, and molecular docking results, it could be concluded that the related mechanisms of minocycline against SCI through anti-oxidative stress, anti-inflammation, and anti-apoptosis (Fig. **12**). Minocycline showed its anti-neuroinflammatory properties *via* inhibiting microglia activation, reducing the pro-inflammatory cytokines including IL-6, TNF-α. At the same time, minocycline reduced the MDA level in the injured spinal cord and increased the SOD level after SCI. In short, minocycline played an important role in anti-inflammatory, anti-apoptotic, and anti-oxidative stress through PI3K/Akt, chemokine, and MAPK signaling pathways after SCI.

### 4.6 Strength and Limitation

This meta-analysis is the first review focusing on minocycline treating SCI, and the final results showed the anti-oxidative stress, anti-inflammation, and anti-apoptosis of minocycline. In addition, we summarized the related signaling pathways (Table **7**) and key targets of minocycline against SCI through network pharmacology and molecular docking approach. However, there are limitations to this research. Although we carefully searched pertinent papers, including some unpublished grey literature and conference proceedings, and searched both Chinese and English databases, we might have missed some articles owing to database restrictions. In addition, the included literature's level of quality was unsatisfactory, and some of it had little clinical significance and value, which may have caused bias in the analysis. BBB score is the main assessment method to evaluate neurological and functional recovery after SCI. Although it is very subjective and depends on fabricated observation and interpretation, bias is simple to introduce. In subgroup analysis, the group that changed the drug dose showed significant differences. However, the specific dose of minocycline is different among different articles, which is another crucial element in determining the analysis's findings. Furthermore, due to significant physiological differences between humans and rats, we would further explore clinical trials to investigate the effectiveness of minocycline in the human body.

## CONCLUSION

This is the first meta-analysis with network pharmacology and molecular docking approach studying minocycline treating SCI. Minocycline can promote nerve recovery after SCI, inhibit inflammation and oxidative stress, and reduce apoptosis. The result of this article should be interpreted and used with the proper level of caution because of the need to apply further creative research, which may overstate the effectiveness of minocycline. Nevertheless, minocycline may one day prove to be a successful treatment for SCI. It merits a more thorough investigation that incorporates the design aspects suggested in this meta-analysis.

## Figures and Tables

**Table 1:** Description of characteristics of the included studies in the meta-analysis.

**Study**	**Animals**	**SCI**	**Experimental Groups**	**Control Group**	**Outcome**
Cang BC 2015 [15]	Rat	T8-T9 spinal cord compression	SCI+MINO (45 mg/kg i.p.)	SCI+NS	Motor function: BBB Histology: HE staining (damage area); The dry-wet mass ratio of spinal cord tissue
Duan BY 2013 [16]	Rat	K-wires hitting the spinal cord	SCI+MINO (Not mentioned)	SCI+NS	Motor function: BBB Biomedical findings: expression of BDNF and NT-3
Takeda M 2011 [17]	Rat	descending aorta clipping and spinal cord compression	SCI+MINO (45 mg/kg in first 36 hours and thereafter 22.5 mg/kg i.p.)	SCI+NS	Motor function: BBB Histology: The percentage of vacuolations in the white matter; The numbers of normal neurons Histology: The number of Iba-1 positive cells
Feng XB 2012 [18]	Rat	T10 weight-drop contusion injury	SCI+MINO (80 mg/kg i.p.)	A: SCI+NS B: SCI+MINO (40 mg/kg i.p.)	Motor function: BBB Histology: Patho-morphologic changes Biomedical findings: The expression of GFAP
Wang G 2006 [19]	Rat	T10-T12 weight-drop contusion injury	SCI+MINO (90 mg/kg i.p.)	SCI+NS	Motor function: BBB Biomedical findings: Cytochrome C immune histochemical detection; percentage of apoptotic cells
Huang XWY 2019 [20]	Rat	T12-T13 spinal cord compression	SCI+MINO (93.75 mg (D1)/68.75 mg (D2)/25 mg (D3), 2 ml/kg, i.p.)	SCI+NS	Motor function: BBB Histology: LFB staining; HE staining (damage area) Others: Motor evoked potential; sensory evoked potential;
Zhao QL 2022 [21]	Rat	T9 spinal cord weight-drop contusion injury	SCI+MINO (6.4mg/kg i.p.)	SCI+NS	Motor function: BBB Biomedical findings: Expression of GPX4 and xCT; RT-qPCR test; Histology: spinal cord morphological changes Others: The percentage of NO
Pinzon A 2008 [22]	Rat	T9-T10 weight-drop contusion injury	SCI+MINO (90 mg/kg immediately after surgery and 45 mg/kg/IP at 12 h and 24 h i.p.)	SCI+NS	Motor function: BBB Histology: the area of spared tissue and cavity
Tan AM 2009 [23]	Rat	T9 weight-drop contusion injury	SCI+MINO (90 mg/kg 5 minutes after SCI for 5 additional days, 45 mg/kg twice daily i.p.)	SCI+NS	Motor function: BBB Pain: Mechanical nociceptive thresholds; Thermal paw-withdrawal latency Histology: the presence of microglia; evoked responses to peripheral stimuli
Festoff BW 2006 [24]	Rat	T9 weight-drop contusion injury	SCI+MINO (90 mg/kg i.p.)	A: SCI+MINO (30 mg/kg i.p.) B: SCI+NS C: SCI+ tetracycline	Motor function: BBB Histology: neuronal profiles; cavity size Immunohistochemical findings: apoptosis; caspase-3 expression; Microglial profiles; TNF-α mRNA levels
Stirling DP 2004 [25]	Rat	C7-C8 dorsal column transection	SCI+MINO (50 mg/kg i.p.)	SCI+NS	Motor function: hindlimb angle of rotation Histology: degenerating white matter; lesion size; active caspase-3-mediated oligodendrocyte death; ED1 density; CST dieback
Schmidt EKA 2021 [26]	Rat	C5 weight-drop contusion injury	SCI+MINO (50 mg/kg PO.)	A: SHAM B: SHAM+MINO C: SCI+NS	Motor function: analysis of the distance traveled Histology: lesion size; microglial density and morphology Immunohistochemical findings: inhibition of inflammatory cytokines; anxiety-like behavior; forepaw asymmetry; Sucrose preference test; fecal microbiota
Sonmez E 2013 [27]	Rat	T10 weight-drop contusion injury	SCI+MINO (90 mg/kg immediately after the trauma and 45 mg/kg for 1 day i.p.)	A: SHAM B: SHAM+NS C: SCI+MINO (1 mg/kg i.v. *via* tail vein)	Motor function: BBB Histology: spinal cord ultrastructure; Apoptotic Index Biomedical findings: analysis of malondialdehyde (MDA) and glutathione (GSH).
Squair JW 2018 [28]	Rat	T3 weight-drop contusion injury	SCI+MINO (90mg/kg after injury, then 45mg/kg every 12 hours for two weeks, i.p.)	SCI+NS	Motor function: BBB; Histology: lesion area, descending sympatho-excitatory axons
Saganová K 2008 [29]	Rat	T10 balloon-compression	SCI+MINO (90 mg/kg at 1 h post-SCI, then 45 mg/kg, every subsequent 12 h for 5 days i.p.)	A: SHAM B: SHAM+MINO (for 1 day)	Motor function: BBB; Histology: spared white/grey matter Others: rats’ weight
Afshary K 2020 [30]	Rat	T9 compression with an aneurysmal clip	SCI+MINO (90 mg/kg minocycline after the operation and 45 mg/kg every 12 h i.p.)	A: SHAM B: SCI+NS C: SCI+pretreatment	Motor function: BBB; Pain: Thermal Allodynia; von Frey filaments test Inflammatory Cytokines Assessment: TNF-α, IL-6 and IL-1β Histology: cumulative histopathologic scores
Lee SM 2003 [31]	Rat	T9-T10 weight-drop contusion injury	SCI+MINO (90 mg/kg immediately after SCI and then 45 mg/kg every 12 h i.p.)	SCI+NS	Motor function: BBB; Histology: Lesion Area Analysis Others: Caspase-3 Activity; apoptotic cell death; IL-10 and TNF-a expression
Sencar L 2020 [32]	Rat	T2-T7 extradural clip compression for 1 minute	SCI+MINO (Initial 90 mg/kg and then 50 mg/kg/day i.v.)	A: Control B: SHAM C: SCI D: SCI+MP E: SCI+MINO+MP	Histology: edematous and hemorrhagic areas Immunohistochemical findings: TNF-α and IL-6; Biomedical findings: MDA and SOD levels;
Aras M 2015 [33]	Rat	T8–T10 weight-drop contusion injury	SCI+MINO (90 mg/kg at the first and 24^th^ hours after SCI po.)	A: SHAM B: SCI C: SCI + MINO (3mg/kg po.) D: SCI + MINO (30 mg/kg po.)	Biomedical findings: MDA, SOD, GSH-Px, TAS, TOS, AST and LDH levels
Teng YD 2004 [34]	Rat	T10 weight-drop contusion injury	SCI+MINO (90 mg/kg 1 h after injury and then 45 mg/kg every 12 h for 5 consecutive days i.p.)	SCI+NS	Motor function: BBB; inclined plane performance Immunohistochemical findings: Cytochrome c Histology: lesion volume Biomedical findings: GFAP and CNPase
Ueno T 2011 [35]	Rat	T10 weight-drop contusion injury	SCI+MINO (Initial dose of 90 mg/kg, then 9 h later 45 mg/kg twice a day up to 3 days post-injury i.p.)	SCI+NS	Motor function: BBB; Biomedical findings: NF-H (pNF-H)
Xu J 2021 [36]	Rat	T9 weight-drop contusion injury	SCI+MINO (50 mg/kg 1 h and 24 h after injury, followed by 25 mg/kg daily for 5 days i.p.)	A: Control B: SCI	Motor function: BBB; tiltboard scores Biomedical findings: MDA, SOD Immunohistochemical findings: GFAP; BDNF
Yune TY 2007 [37]	Rat	T9-T10 weight-drop contusion injury	SCI+MINO (90 mg/kg immediately and 2 h after injury, and then 45 mg/kg every 12 h for 3 days i.p.)	A: SCI+NS B: SCI+ MP	Motor function: BBB; angles of inclined plane Immunohistochemical findings: proNGF; p75 neurotrophin receptor expression and RhoA; caspase-3 staining
Zhang G 2019 [38]	Rat	T9-T10 weight-drop contusion injury	SCI+MINO (90 mg/kg and later a 45 mg/kg dose twice a day for 2 weeks, i.p.)	A: Control B: SCI+ NS	Immunohistochemical findings: Smac/Diablo, cytochrome-c, HIF-1α, FAS ligand; TNF-α

**Abbreviations**: BBB scale, Basso, Beattie, and Bresnahan scale; MINO, minocycline; h, hour(s); *i.p.*, intraperitoneally; SCI, Spinal cord injury; NS, normal saline; *i.v*., intravenous; *po.*, peros; SD, Sprague-Dawley; min, minute(s); s, second; h, hour; d(s), day(s); BDNF, brain-derived neurotrophic factor; MDA, malondialdehyde; GSH, glutathione; TNF-α, tumour necrosis factor-α; SOD, superoxide dismutase; GFAP, glial fibrillary acidic protein; Iba-1, ionized calcium binding adaptor molecule 1; GPX4, glutathione peroxidase 4; xCT, cystine/glutamate antiporter system; NO, nitrous oxide; NT-3, neurotrophin-3; ED1, lysosomal monocyte/macrophage antigen; IL-6, interleukin-6; IL-1β, interleukin-1β; IL-10, interleukin-10; TAS, total antioxidant status; TOS, total antioxidant status; LDH, lactate dehydrogenase; AST, aspartate aminotransferase; CNPase, 2’,3’Cyclic Nucleotide 3’Phosphodiesterase; pNF-H, phosphorylated neurofilament H; proNGF, pro-nerve growth factor; RhoA, ras homolog gene family member A; HIF-1α, Hypoxia-Inducible Factor 1.

**Table 2:** Risk of bias.

**References**	**1**	**2**	**3**	**4**	**5**	**6**	**7**	**8**	**9**	**10**	**Score**
Afshary K 2020	+	+	+	?	?	+	+	?	+	+	**7**
Aras M 2015	+	+	+	?	?	+	+	?	+	+	**7**
Cang BC 2015	+	?	+	?	+	+	+	?	+	?	**6**
Duan BY 2013	+	?	+	?	?	+	+	?	+	?	**5**
Feng XB 2012	+	?	+	?	+	+	+	?	+	?	**6**
Festoff BW 2006	+	+	+	?	?	+	+	?	+	?	**6**
Huang XWY 2019	+	?	+	?	?	+	+	?	+	?	**5**
Lee SM 2003	+	+	+	?	+	+	+	?	+	?	**7**
Pinzon A 2008	+	+	+	?	?	+	+	?	+	?	**5**
Saganová K 2008	+	?	+	?	?	+	+	?	+	?	**5**
Schmidt EKA 2021	+	+	+	?	+	+	+	?	+	+	**8**
Sencar L 2020	+	?	+	?	?	+	+	?	+	+	**6**
Sonmez E 2013	+	+	+	?	?	+	+	?	+	?	**6**
Squair JW 2018	+	?	+	+	+	+	+	?	+	+	**8**
Stirling DP 2004	+	+	+	?	?	+	+	?	+	?	**6**
Takeda M 2011	+	?	+	?	+	+	+	?	+	?	**6**
Tan AM 2009	+	+	+	?	+	+	+	?	+	?	**7**
Teng YD 2004	+	?	+	?	?	+	+	?	+	?	**5**
Ueno T 2011	+	?	+	?	?	+	+	?	+	+	**6**
Wang G 2006	+	?	+	?	?	+	+	?	+	?	**5**
Xu J 2021	+	?	+	?	+	+	+	?	+	+	**7**
Yune TY 2007	+	?	+	?	?	+	+	?	+	?	**5**
Zhang GL 2019	+	+	+	?	?	+	+	?	+	+	**7**
Zhao QL 2022	+	+	+	?	+	+	+	?	+	?	**7**

**Note**: (1) peer-reviewed publication; (2) control of temperature; (3) random allocation to treatment or control; (4) blinded induction of ischemia; (5) blinded assessment of outcome; (6) use of anesthetic without significant intrinsic neuroprotective activity; (7) animal model (aged, diabetic, or hypertensive); (8) sample size calculation; (9) compliance with animal welfare regulations; and (10) statement of potential conflict of interests.

**Table 3:** Subgroup analysis of minocycline statistical data.

**Subgroup**	**No. of Studies**	**MD (95% CI)**	**Heterogeneity**		**Subgroup Difference**
			***I^2^***	***p-value***	
*Modeling* Contusion Compression	11 3	2.79 (1.58, 4.01) 0.85 (-1.51, 3.21)	*98* *91*	*p<0.00001* *p<0.00001*	*p = 0.15*
*Segment* T9-T10 T10-T13	10 2	2.52 (1.12, 3.92) 1.48 (-1.61, 4.57)	*99* *98*	*p<0.00001* *p<0.00001*	*p = 0.55*
*Dosage* 90 mg/kg 45 mg/kg	9 2	2.50 (1.04, 3.96) 11.87 (1.23, 22.51)	*99* *99*	*p<0.00001* *p<0.00001*	*p = 0.09*
*Mode of drug dose* Multi Once	9 4	1.74 (1.27, 3.54) 4.07 (2.76, 5.37)	*99* *84*	*p<0.00001* *p=0.0004*	*p = 0.02*
*Placebo* Saline Untreated	10 3	2.41 (1.27, 3.54) 2.44 (0.63, 4.25)	*98* *98*	*p<0.00001* *p<0.0000*	*p = 1.00*
*Study Quality* High Low	7 6	3.52 (2.61, 4.42) 2.41 (1.27, 3.54)	*94* *98*	*p<0.00001* *p<0.00001*	*p = 0.001*

**Table 4:** Biochemical outcomes of minocycline treating SCI.

**Biochemical Outcomes**	**No. of Studies**	**SMD (95% CI)**	***P-*value**
MDA	3	(-7.55, -2.01)	*p* = 0.002
SOD	2	(4.11, 8.49)	*p* = 0.72
IL-6	2	(-16.53, -0.80)	*p<*0.0001
TNF-α	4	(-8.61, -1.38)	*p<*0.0001

**Table 5:** KEGG signaling pathways and correspondent target genes related to SCI from minocycline.

**Pathway**	**Count**	**Genes**
Pathways in cancer	26	AKT1, ALK, BAD, BCL2, BCL2L1, CTNNB1, EGFR, ERBB2, ESR1, GSK3B, HSP90AA1, IKBKB, KIT, MMP1, NOS2, PIK3CA, PRKACA, PRKCA, PRKCB, PRKCG, PTGS2, RARA, RELA, HSP90B1, ROCK2, EGLN1
Lipid and atherosclerosis	17	AKT1, BAD, BCL2, BCL2L1, ERN1, GSK3B, HSPA1A, HSPA8, HSP90AA1, IKBKB, MMP1, PIK3CA, PRKCA, RELA, SRC, HSP90B1, ROCK2
Human cytomegalovirus infection	16	AKT1, CCR5, CTNNB1, EGFR, GSK3B, IKBKB, CXCR2, PIK3CA, PRKACA, PRKCA, PRKCB, PRKCG, PTGS2, RELA, SRC, ROCK2
Chemokine signaling pathway	15	AKT1, BAD, CCR4, CCR5, GSK3B, IKBKB, CXCR2, PIK3CA, PRKACA, PRKCB, PRKCD, PRKCZ, RELA, SRC, ROCK2
PI3K-Akt signaling pathway	15	AKT1, BAD, BCL2, BCL2L1, EGFR, ERBB2, GSK3B, HSP90AA1, IKBKB, KIT, MCL1, PIK3CA, PRKCA, RELA, HSP90B1
Chemical carcinogenesis - receptor activation	15	AKT1, BAD, BCL2, EGFR, ESR1, HSP90AA1, PIK3CA, PRKACA, PRKCA, PRKCB, PRKCG, RELA, RPS6KA3, SRC, HSP90B1
Estrogen signaling pathway	14	AKT1, BCL2, EGFR, ESR1, HSPA1A, HSPA8, HSP90AA1, OPRM1, PIK3CA, PRKACA, PRKCD, RARA, SRC, HSP90B1, BAD, PRKCA, PRKCB, PRKCG, RELA, RPS6KA3, ERBB2, BCL2L1, ERN1, MCL1, MPL, NOS2, TLR9
MicroRNAs in cancer	14	BCL2, EGFR, ERBB2, IKBKB, MCL1, ABCB1, PIK3CA, PLAU, PRKCA, PRKCB, PRKCE, PRKCG, PTGS2, SIRT1
Prostate cancer	13	AKT1, BAD, BCL2, CTNNB1, EGFR, ERBB2, GSK3B, HSP90AA1, IKBKB, PIK3CA, PLAU, RELA, HSP90B1
Focal adhesion	13	AKT1, BAD, BCL2, CTNNB1, EGFR, ERBB2, GSK3B, PIK3CA, PRKCA, PRKCB, PRKCG, SRC, ROCK2
Proteoglycans in cancer	13	AKT1, CTNNB1, EGFR, ERBB2, ESR1, PIK3CA, PLAU, PRKACA, PRKCA, PRKCB, PRKCG, SRC, ROCK2
Human immunodeficiency virus 1 infection	13	AKT1, BAD, BCL2, BCL2L1, CDK1, CHEK1, CCR5, IKBKB, PIK3CA, PRKCA, PRKCB, PRKCG, RELA
MAPK signaling pathway	13	AKT1, EGFR, ERBB2, HSPA1A, HSPA8, IKBKB, KIT, PRKACA, PRKCA, PRKCB, PRKCG, RELA, RPS6KA3
Pathways of neurodegeneration - multiple diseases	13	BAD, BCL2, BCL2L1, CTNNB1, ERN1, GSK3B, NOS2, PRKCA, PRKCB, PRKCG, PTGS2, RELA, NOX4
Shigellosis	12	AKT1, BCL2, BCL2L1, EGFR, GSK3B, IKBKB, PIK3CA, PRKCD, PRKCE, RELA, SRC, ROCK2
Alzheimer disease	12	AKT1, BAD, CTNNB1, ERN1, GSK3B, IKBKB, NOS2, PIK3CA, PTGS2, RELA, BACE1, NOX4

**Abbreviations**: KEGG, Kyoto Encyclopedia of Genes and Genomes; SCI, spinal cord injury.

**Table 6:** Docking energy.

**Protein**	**Spacing (Angstrom)**	**Grid Box**	**Energy**	**H Bonds**
2uzp	0.774	98*98*102	-4.58	0
4u5j	0.786	82*98*114	-3.47	2
6fbx	0.353	126*126*126	-5.35	5
ALOX12	0.747	126*126*126	-3.79	2
ALOX15	0.992	126*126*126	N/A	N/A
BAD	0.714	58*70*84	-3.37	2
BCL2L1	0.714	104*70*84	-3.69	2
CDK1	0.769	104*126*126	-3.66	2
CHEK1	0.769	76*90*106	-4.3	2
CHEK2	0.769	116*90*106	-5.0	1
CTNNB1	0.425	98*78*82	-6.54	2
CXCR2	0.569	126*126*126	-3.98	1
EGFR	0.569	106*126*126	-4.7	2
ERN1	1.00	126*80*126	-2.53	0
GSK3B	0.728	110*126*126	-3.35	0
HSP90AA1	0.728	126*126*126	-5.07	2
IKBKB	0.856	86*86*126	-2.72	3
NOS2	0.700	86*98*102	-3.7	3
NOX4	0.811	90*98*126	-4.25	1
PLAU	0,478	90*104*84	-6.62	2
PRKACA	0.533	98*126*104	-6.87	1
PRKCA	0.533	98*126*80	-4.59	1
PRKCB	0.739	126*126*102	-4.95	1
PRKCD	0,339	126*126*114	-4.17	2
PRKCZ	0.992	126*126*126	N/A	N/A
PTGS1	0.844	126*98*98	-3.21	2
PTGS2	1.00	126*98*126	N/A	N/A
RARA	0.583	126*114*126	-3.92	1
RELA	0.522	104*114*126	-4.02	0
ROCK2	0.900	82*114*102	-3.41	1

**Table 7:** Summary of signal pathways and mechanisms.

**References**	**Mechanism**	**Pathway**
Cang BC 2015 [15]	Inflammation	Caspase-3
Duan BY 2013 [16]	Inflammation	BDNF, NT-3
Takeda M 2011 [17]	Inflammation, Apoptosis	Iba-1
Feng XB 2012 [18]	Inflammation	GFAP
Wang G 2006 [19]	Apoptosis	Cytochrome C
Huang XWY 2019 [20]	Inflammation	/
Zhao QL 2022 [21]	Oxidative stress, Inflammation	GPX4, xCT
Pinzon A 2008 [22]	Inflammation	/
Tan AM 2009 [23]	Apoptosis	PGE2
Festoff BW 2006 [24]	Inflammation, Apoptosis	Caspase-3, TNF-α
Stirling DP 2004 [25]	Apoptosis	Caspase-3, GFAP
Schmidt EKA 2021 [26]	Inflammation	Eotaxin, EGF, Fractalkine, IFN-gamma, IL-1a, IL-1b, IL-2, IL-4, IL-5, IL-6, IL-10, IL-12(p70), IL-13, IL-17A, IL-18, IP-10, GRO/KC, TNF-α, G-CSF, GM-CSF, MCP-1, Leptin, LIX, MIP-1alpha, MIP-2, RANTES, VEGF, corticosterone, melatonin
Sonmez E 2013 [27]	Inflammation, Apoptosis	MDA, GSH
Squair JW 2018 [28]	Inflammation	/
Saganová K 2008 [29]	Inflammation	/
Afshary K 2020 [30]	Inflammation	TNF-α, NO, IL-6, IL-1β
Lee SM 2003 [31]	Inflammation, Apoptosis	TNF-α, IL-10, Caspase-3
Sencar L 2020 [32]	Inflammation, Oxidative stress	TNF-α, IL-6, MDA, SOD
Aras M 2015 [33]	Oxidative stress	SOD, MDA, GSH-Px, TAS, TOS, LDH, AST
Teng YD 2004 [34]	Inflammation, Apoptosis	Cytochrome c, GFAP, CNPase
Ueno T 2011 [35]	Inflammation	NF-H (pNF-H)
Xu J 2021 [36]	Inflammation, Apoptosis, Oxidative stress	MDA, SOD, GFAP, BDNF
Yune TY 2007 [37]	Inflammation, Apoptosis	proNGF, p75, RhoA, Caspase-3
Zhang G 2019 [38]	Apoptosis	Cytochrome-c, HIF-1α, FAS ligand, TNF-α

## Data Availability

The corresponding author would provide the datasets used and analyzed during the current work upon reasonable request.

## LIST OF ABBREVIATIONS

CNS: Central Nervous System
CTNNB1: Catenin Cadherin-associated Protein-beta 1
EGFR: Epidermal Growth Factor Receptor
GFAP: Glial Fibrillary Acidic Protein
IL-6: Interleukin-6
MCP-1: Monocyte-chemoattractant Protein-1
MDA: Malondialdehyde
ROS: Reactive Oxygen
SCI: Spinal Cord Injury
SOD: Superoxide Dismutase
SRC: Steroid Receptor Coactivator
TNF-α: Tumor Necrosis Factor-α

## STANDARDS OF REPORTING

PRISMA guidelines and methodology were followed.

## SUPPLEMENTARY MATERIAL

PRISMA checklist is available as supplementary material on the publisher’s website along with the published article.
